# Hearing Loss in Id1^−/−^; Id3^+/−^ and Id1^+/−^; Id3^−/−^ Mice Is Associated With a High Incidence of Middle Ear Infection (Otitis Media)

**DOI:** 10.3389/fgene.2021.508750

**Published:** 2021-08-09

**Authors:** Qingyin Zheng, Tihua Zheng, Aizhen Zhang, Bin Yan, Bo Li, Zhaoqiang Zhang, Yan Zhang

**Affiliations:** ^1^Department of Otolaryngology – Head and Neck Surgery, Second Affiliated Hospital of Xi’an Jiaotong University School of Medicine, Xi’an, China; ^2^School of Medicine, Case Western Reserve University, Cleveland, OH, United States; ^3^College of Special Education, Hearing and Speech Rehabilitation Institute, Binzhou Medical University, Yantai, China

**Keywords:** otitis media, mouse model, hearing loss, inflammation, genetic predisposition

## Abstract

Inhibitors of differentiation/DNA binding (Id) proteins are crucial for inner ear development, but whether Id mutations affect middle ear function remains unknown. In this study, we obtained Id1^−/−^; Id3^+/−^ mice and Id1^+/−^; Id3^−/−^ mice and carefully examined their middle ear morphology and auditory function. Our study revealed a high incidence (>50%) of middle ear infection in the compound mutant mice. These mutant mice demonstrated hearing impairment starting around 30 days of age, as the mutant mice presented elevated auditory brainstem response (ABR) thresholds compared to those of the littermate controls. The distortion product of otoacoustic emission (DPOAE) was also used to evaluate the conductive function of the middle ear, and we found much lower DPOAE amplitudes in the mutant mice, suggesting sound transduction in the mutant middle ear is compromised. This is the first study of the middle ears of Id compound mutant mice, and high incidence of middle ear infection determined by otoscopy and histological analysis of middle ear suggests that Id1/Id3 compound mutant mice are a novel model for human otitis media (OM).

## Introduction

Inhibitors of differentiation/DNA binding (Id) proteins are major inhibitors of helix–loop–helix (HLH) transcription factors. The Id genes encode four related transcription factors: Id1, Id2, Id3, and Id4. Id transcription factors contain a helix-loop-helix region similar to that of the basic helix–loop–helix (bHLH) transcription factors, can form heterodimers with some of them and lose their binding to DNA. However, due to the lack of basic DNA-binding regions, these heterodimers cannot bind to DNA. Consequently, Id transcription factors negatively regulate the DNA binding capacity of bHLH proteins (Benezra et al., 1990; Wong et al., 2004). In other words, Id transcription factors usually hinder cell differentiation, especially the differentiation of dendritic cells, which plays a rather important role in immunity to infectious agents (Spits et al., 2000; Kee, 2009).

Inhibitors of differentiation/DNA binding proteins regulate different kinds of cellular processes, including cellular growth, differentiation, migration, senescence, and tumorigenesis (Sikder et al., 2003; Wong et al., 2004; Wu et al., 2018). Id1, Id2, and Id3 are expressed in the otic vesicle of mouse embryos (Jen et al., 1997). The importance of these three Id genes as differentiation regulators was also illustrated by their key role in the regulation of expression of Math1 and hair cell differentiation in the developing cochlea (Jones et al., 2006). Ids expression profiles in the normal mature middle ear has not been reported. However, a previous study by Lin et al. (2002) showed that Id1 and Id3 are unregulated in the middle ears of rats following pneumococcal infection, which indicates that Id1 and Id3 may be involved in middle ear diseases such as otitis media (OM). The main objective of this study was to clarify the correlation between the two Id genes with OM.

The most common middle ear problems include cholesteatoma, tympanic membrane (TM) perforation, middle ear infection, and otosclerosis. Among these diseases, middle ear infection, or OM, is the most common, as most children younger than 3 years will have at least one episode of OM (McCaig et al., 2002; O’Brien et al., 2009). Acute OM is the most common cause of meningitis, whereas neglected chronic OM can lead to permanent hearing loss (Pont and Mazon, 2017). Previous studies have shown that genetic lesions lead to a high incidence of OM in mutant animals such as Jeff mice, Sh3pxd2b mice, and Enpp1asj mice (Hardisty-Hughes et al., 2006; Yang et al., 2011; Tian et al., 2016). Animal models with OM susceptible to a defined genetic lesion will be important to reveal the pathogenesis and underlying genetic pathways linked to OM.

Individual null mutants for Id1, Id2, and Id3 have been generated and reported to have very few abnormalities, and none associated with hearing deficit (Yan et al., 1997; Lyden et al., 1999; Yokota et al., 1999). Id1 and Id3 double-knockout mice are commonly used as animal models to study the role of Id1 and Id3 in mammalian development (Zhao et al., 2011). Unfortunately, complete loss of these genes leads to aggregation of dilated and irregularly shaped blood vessels and brain hemorrhage by E12.5, and no embryos survived beyond E13.5 (Lyden et al., 1999; Fraidenraich et al., 2004). Because of the upregulated expression of Id1 and Id3 in middle ears after the acute episode of pneumococcal otitis media has been resolved, the two genes are presumed to be involved in the development of otitis media (Lin et al., 2002). In this study, the auditory brainstem response (ABR) and middle ear morphology in Id1^−/−^; Id3^+/−^ and Id1^+/−^; Id3^−/−^ mice were carefully examined. OM and associated elevated hearing thresholds were found in the majority of the mutant mice. Our data suggest that Id1/Id3 mice are valuable models for the study of OM pathogenesis and associated genetic factors.

## Materials and Methods

### Mice and Animal Care

Our Id1 and Id3 single-knockout mice were generous gifts from Dr. Robert Benezra. Id1^−/−^; Id3^+/−^ or Id1^+/−^; Id3^−/−^ mice were generated by intercrossing double heterozygous mice. Littermate mice with Id1^+/−^; Id3^+/−^, Id1^+/+^; Id3^+/−^, Id1^+/−^; Id3^+/+^ or Id1^+/+^; Id3^+/+^ genotypes were generated incidentally during breeding. We used littermate mice with Id1^+/+^; Id3^+/+^ genotype as wild-type controls. Mice of the other three genotypes were excluded because they did not show significant hearing loss (data not shown). No gender difference was found during the experiment. Both male and female mice from the same litter were included as the experimental and control groups. A total of 101, Id1^−/−^; Id3^+/−^ mice, 28 Id1^+/−^; Id3^−/−^ mice and 30 wild-type mice were used in this study. ABR test was performed at four time points: P30 (P: postnatal, same meaning as below), P60, P90, and P120. After ABR test, distortion product of otoacoustic emission (DPOAE) measurement was performed at two time points: P30 and P60. After ABR and DPOAE test at P30, otoscopic examination was carried out and then some of the mice were euthanized to conduct Hematoxylin-Eosin (H&E) staining. Only the data of the right ear were counted in the analysis of the experimental results. Specific details on the use of mice can be found in the Supplementary Figure 1. The experimental protocols were approved by the Case Western Reserve University Animal Care and Use Committee and were in agreement with the National Institutes of Health Guide for the Care and Use of Laboratory Animals. Studies were conducted according to the principles set forth in the Guide for the Care and Use of Laboratory Animals (DC005846) as well as the Institute of Laboratory Animal Resources (protocol 2014-0155).

### Mice Genotyping

Genomic DNA was obtained from mouse tail tips. PCR analyses were performed with primers specific for the wild-type and targeted alleles. Primer sequences for Id1 were ID1F1 (wild-type oligonucleotide; 5ꞌ-TCCTGCAGCATGTAATCGAC-3ꞌ), ID1F2 (mutant oligonucleotide; 5ꞌ-GACACCCACTGGAAAGGACA-3ꞌ), and ID1R1 (common oligonucleotide; 5ꞌ-GAGACCCACTGGAAAGGACA-3ꞌ). Primers sequences for Id3 were ID3F1 (wild-type oligonucleotide; 5ꞌ-CTTGGGACCCTGGGACTCT-3ꞌ), ID3F2 (mutant oligonucleotide; 5ꞌ-GGGGAACTTCCTGACTAGGG-3ꞌ), and ID3R1 (common oligonucleotide; 5ꞌ-TAATCAGGGCAGCAGAGCTT-3ꞌ). The amplified PCR products were analyzed on 2% agarose gels to separate the wild-type (344 bp for ID1 and 350 bp for ID3) and targeted allele (540 bp for ID1 and 500 bp for ID3) fragments.

### Auditory Brainstem Response, Distortion Product Otoacoustic Emission Tests, and Otoscopic Examination

Auditory brainstem response threshold analyses were carried out using equipment from Intelligent Hearing Systems (Miami, FL, United States) as previously described (Zheng et al., 1999). Prior to examination, mice were anesthetized with 2, 2 and 2-tribromoethanol (Avertin; 0.5 mg/g body mass), and their body temperature was kept at 37–38°C by placing them on a heating pad in a soundproof chamber during testing. Specific auditory stimuli (broadband click and pure-tone burst stimuli of 8, 16, and 32 kHz) from ER2 and high-frequency transducers were delivered through plastic tubes to the ear canals. Evoked brainstem responses were amplified and averaged, and their wave patterns were displayed on a computer screen. Auditory thresholds were obtained for each stimulus by varying the SPL in 10 dB steps and, finally, a 5 dB step up and down to identify the lowest level at which an ABR pattern could be recognized.

The DPOAE test was performed using SmartOAE 4.50 USBez software (Intelligent Hearing Systems) and an Etymotic 10B+ OAE probe (Etymotic Research, Elk Grove Village, IL) fitted with a high-frequency transducer (Intelligent Hearing Systems) that produced two pure tones, F1 and F2, respectively. The methods and process followed those described in a previous paper (Zhang et al., 2012).

After the ABR and DPOAE tests, otoscopy was performed on both ears to determine the condition of TM, including presence of middle ear fluid, inflammation, or infection.

### Histological Analysis of Middle Ear

Ten Id1/Id3 heterozygous (Id1^−/−^; Id3^+/−^ and Id1^+/−^; Id3^−/−^) and 10 wild-type mice (129Sv/C57BL6) aged 30 days of age were euthanized and decapitated. Both bullae, including the middle and inner ear (IE), were rapidly removed. The bullae were then fixed in 4% PFA, decalcified with 10% EDTA solution, dehydrated and embedded in paraffin. The paraffin block was serially sectioned at 5-μm thicknesses and stained with H&E for light microscopic examination.

### Statistical Analysis

The data are presented as mean ± SEM. The difference between groups was determined using a one-way or two-way ANOVA or unpaired student *t*-test when applicable using GraphPad Prism software.

## Results

### ABR Thresholds and DPOAE in the Id1^−/−^; Id3^+/−^ and Id1^+/−^; Id3^−/−^ Mice Indicated Hearing Loss in a Majority of Mice

Auditory brainstem response tests were carried out and analyzed with equipment from Intelligent Hearing Systems using previously described methods and equipment (Zheng et al., 1999). Mice with ABR threshold values above 55 (for click stimuli), 40 (for 8 kHz), 35 (for 16 kHz), or 60 (for 32 kHz) dB SPL were considered hearing impaired (Zheng et al., 1999). Of the 101 Id1^−/−^; Id3^+/−^ mice observed, 89.1% (90/101) had high ABR thresholds for at least one stimulus frequency (click, 8, 16, and 32 kHz) in at least one ear, with 82.2% (83/101) for the right ears and 81.2% (82/101) for the left ears. Of the 28 Id1^+/−^; Id3^−/−^ mice observed, 100% (28/28) had high ABR thresholds for at least one stimulus frequency (click, 8, 16, and 32 kHz) in at least one ear, with 92.9% (26/28) for the right ears and 92.9% (26/28) for the left ears.

**Figure 1:**
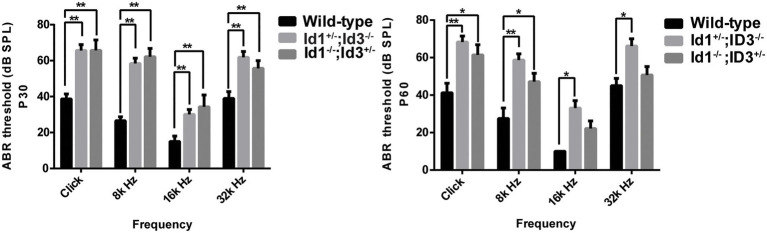
Comparison of the mean auditory brainstem response (ABR) thresholds of right ears from age-matched littermate control mice, Id1^−/−^; Id3^+/−^ and Id1^+/−^; Id3^−/−^ mice at ages P30 and P60. The results indicated that at P30, the mean ABR thresholds at any stimulus frequencies in the Id1^−/−^; Id3^+/−^ (*n* = 41) and Id1^+/−^; Id3^−/−^ (*n* = 14) mice were significantly higher than those of the controls (*n* = 21; *p* < 0.01). At P60, ABR thresholds at all stimulus frequencies in the Id1^−/−^; Id3^+/−^ mice (*n* = 35) were significantly higher than those of the controls (*n* = 8; *p* < 0.01), and ABR thresholds at Click and 8 k Hz in the Id1^+/−^; Id3^−/−^ mice (*n* = 14) were significantly higher than those of the controls (*p* < 0.05). The error bar indicates SD from the mean of each group. ^*^p < 0.05; ^**^p < 0.01.

A comparison of ABR thresholds in the right ears was made between wild-type, Id1^−/−^; Id3^+/−^ and Id1^+/−^; Id3^−/−^ mice at P30 and P60, respectively. The results indicated that, at P30, the mean ABR thresholds at any stimulus frequency in the Id1^−/−^; Id3^+/−^ (*n* = 41) and Id1^+/−^; Id3^−/−^ (*n* = 14) mice were significantly higher than those of the controls (*n* = 21; click ABR threshold: Wild-type vs. Id1^−/−^; Id3^+/−^, *p* < 0.0001; Wild-type vs. Id1^+/−^; Id3^−/−^, *p* < 0.0001; 8 kHz ABR threshold: Wild-type vs. Id1^−/−^; Id3^+/−^, *p* < 0.0001; Wild-type vs. Id1^+/−^; Id3^−/−^, *p* < 0.0001; 16 kHz ABR threshold: Wild-type vs. Id1^−/−^; Id3^+/−^, *p* = 0.0060; Wild-type vs. Id1^+/−^; Id3^−/−^, *p* = 0.0057; 32 kHz ABR threshold: Wild-type vs. Id1^−/−^; Id3^+/−^, *p* < 0.0001; Wild-type vs. Id1^+/−^; Id3^−/−^, *p* = 0.0183; Figure 1). At P60, ABR thresholds at all stimulus frequencies in the Id1^+/−^; Id3^−/−^ (*n* = 14) mice were significantly higher than those of the wild-type controls (*n* = 21; click ABR threshold: Wild-type vs. Id1^+/−^; Id3^−/−^, *p* = 0.0010; 8 kHz ABR threshold: Wild-type vs. Id1^+/−^; Id3^−/−^, *p* = 0.0001; 16 kHz ABR threshold: Wild-type vs. Id1^+/−^; Id3^−/−^, *p* = 0.0058; 32 kHz ABR threshold: Wild-type vs. Id1^+/−^; Id3^−/−^, *p* = 0.0119), and ABR thresholds for click and 8 k Hz in Id1^−/−^; Id3^+/−^ mice (*n* = 35) were significantly higher than those of the wild-type controls (*n* = 21; click ABR threshold: Wild-type vs. Id1^−/−^; Id3^+/−^, *p* = 0.0386; 8 kHz ABR threshold: Wild-type vs. Id1^+/−^; Id3^−/−^, *p* = 0.0451; Figure 1).

**Figure 2:**
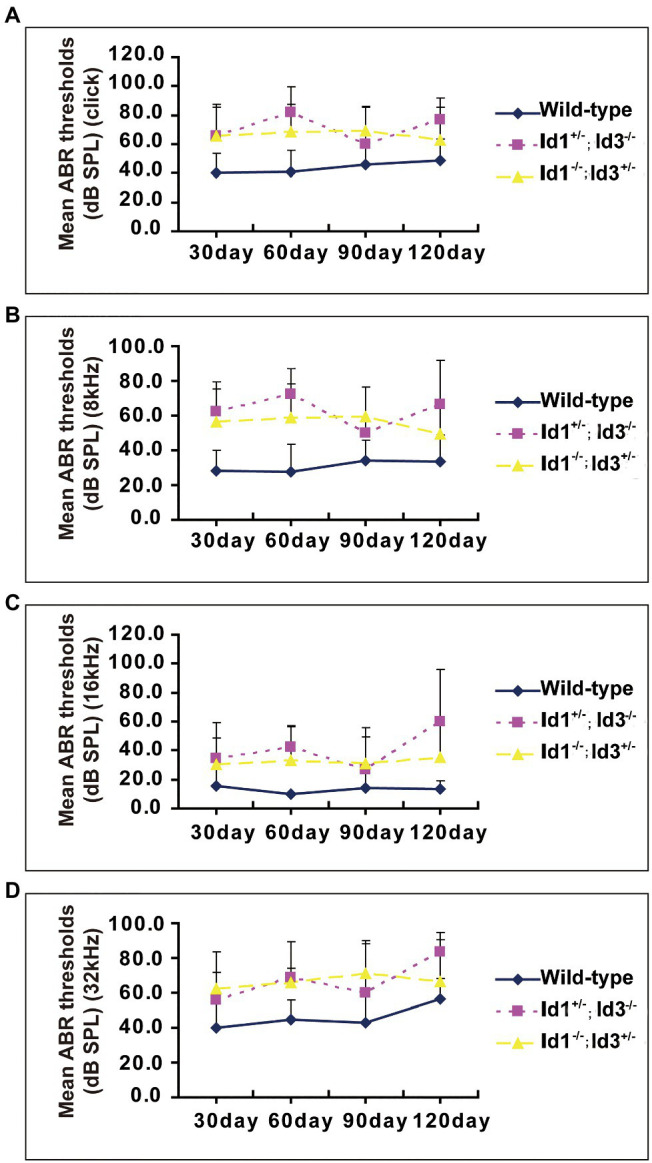
A time-course observation of the ABR thresholds in the right ears of Id1^−/−^; Id3^+/−^ and Id1^+/−^; Id3^−/−^ mice and littermate control mice. All mice of ages from P30 to P120 and the individual ABR thresholds at all stimulus frequencies of click **(A)**, 8 **(B)**, 16 **(C)**, and 32 kHz **(D)** were variable, but the overall tendency was relatively stable at each stimulus frequency compared with the ABR thresholds of the control mice. The error bars indicate SD from the mean at each time point for each mouse group.

A time-course observation of the ABR thresholds in the right ears of Id1/Id3 compound mutant mice and wild-type mice is shown in Figure 2. We divided the mice in each group into four subgroups by approximately 1-month increments in age. The results showed that the mean ABR thresholds of Id1^−/−^; Id3^+/−^ mice (*n* = 41, 35, 31, and 17 in each subgroup, respectively) and Id1^+/−^; Id3^−/−^ mice (*n* = 14, 14, 7, and 6, respectively) were elevated at various time points at all stimulus frequencies. According to the criteria (stated above) for defining hearing loss in mice, Id1/Id3 compound mutant mice present as hearing loss since 30 days at all stimulus frequencies (Figure 2).

**Figure 3:**
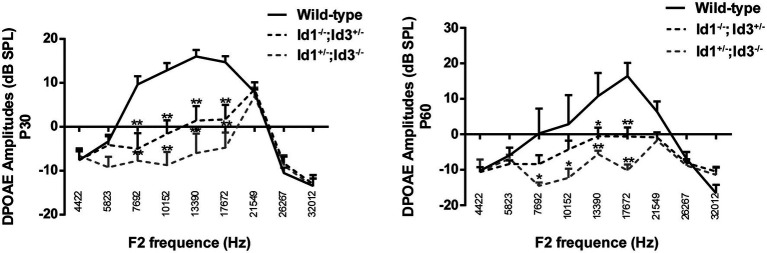
Comparison of the distortion product of otoacoustic emission (DPOAE) amplitudes of right ears from age-matched littermate control mice, Id1^−/−^; Id3^+/−^ and Id1^+/−^; Id3^−/−^ mice at ages P30 and P60. The results indicated that at P30, the DPOAE amplitudes of Id1^−/−^; Id3^+/−^ (*n* = 12) and Id1^+/−^; Id3^−/−^ mice (*n* = 9) were significantly lower than those of the control mice (*n* = 13) at dominant frequencies. At P60, the DPOAE amplitudes at dominant frequencies in the Id1^−/−^; Id3^+/−^ mice (*n* = 35) were significantly lower than those of the controls (*n* = 8), and the DPOAE amplitudes at two frequencies in Id1^+/−^; Id3^−/−^ mice were significantly lower than those of the controls (*n* = 14). The error bar indicates SD from the mean or each group. ^*^p < 0.05; ^**^p < 0.01.

The DPOAE amplitudes were generated from cochlear and recorded in the external auditory canal. DPOAE amplitudes can be influenced by the condition of middle ear. We analyzed the DPOAE amplitudes of right ears from age-matched wild-type mice, Id1^−/−^; Id3^+/−^ and Id1^+/−^; Id3^−/−^ mice at ages of P30 and P60. The results demonstrated that at P30, the DPOAE amplitudes of Id1^−/−^; Id3^+/−^ (*n* = 12) and Id1^+/−^; Id3^−/−^ mice (*n* = 9) at dominant frequencies 7,692, 10,152, 13,390, and 17,672 were significantly lower than those of wild-type mice (*n* = 13; DPOAE amplitude at 7,692: Wild-type vs. Id1^−/−^; Id3^+/−^, *p* < 0.0001; Wild-type vs. Id1^+/−^; Id3^−/−^, *p* < 0.0001; DPOAE amplitude at 10,152: Wild-type vs. Id1^−/−^; Id3^+/−^, *p* < 0.0001; Wild-type vs. Id1^+/−^; Id3^−/−^, *p* < 0.0001; DPOAE amplitude at 13,390: Wild-type vs. Id1^−/−^; Id3^+/−^, *p* < 0.0001; Wild-type vs. Id1^+/−^; Id3^−/−^, *p* < 0.0001; DPOAE amplitude at 17,672: Wild-type vs. Id1^−/−^; Id3^+/−^, *p* < 0.0001; Wild-type vs. Id1^+/−^; Id3^−/−^, *p* < 0.0001). At P60, the DPOAE amplitudes of Id1^−/−^; Id3^+/−^ mice (*n* = 12) at frequencies 13,390, 17,672 were significantly lower than those of wild-type mice (*n* = 3; DPOAE amplitude at 13,390: Wild-type vs. Id1^−/−^; Id3^+/−^, *p* = 0.0344; DPOAE amplitude at 17,672: Wild-type vs. Id1^−/−^; Id3^+/−^, *p* = 0.0009); DPOAE amplitudes of Id1^+/−^; Id3^−/−^ mice (*n* = 6) at dominant frequencies 7,692, 10,152, 13,390, and 17,672 were significantly lower than those of wild-type mice (DPOAE amplitude at 7,692: Wild-type vs. Id1^+/−^; Id3^−/−^, *p* = 0.0137; DPOAE amplitude at 10,152: Wild-type vs. Id1^+/−^; Id3^−/−^, *p* = 0.0112; DPOAE amplitude at 13,390: Wild-type vs. Id1^+/−^; Id3^−/−^, *p* = 0.0057; DPOAE amplitude at 17,672: Wild-type vs. Id1^+/−^; Id3^−/−^, *p* < 0.0001; Figure 3).

### Otitis Media in Id1^−/−^; Id3^+/−^ and Id1^+/−^; Id3^−/−^ Mice

**Figure 4:**
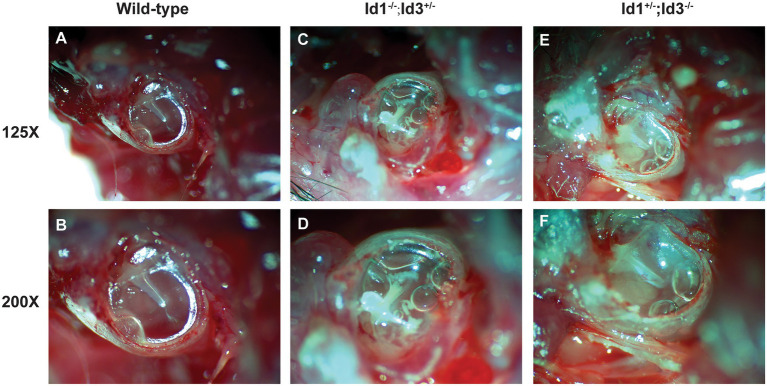
Otoscopic examination of the middle ear in Id1^−/−^; Id3^+/−^ and Id1^+/−^; Id3^−/−^ mice. Compared with the middle ear cavities (MECs) of littermate control mice **(A,B)**, the MECs of Id1^−/−^; Id3^+/−^ mice **(C,D)** and Id1^+/−^; Id3^−/−^ mice **(E,F)** were filled with effusions, and white patches were observed on the tympanic membrane (TM).

**Figure 5:**
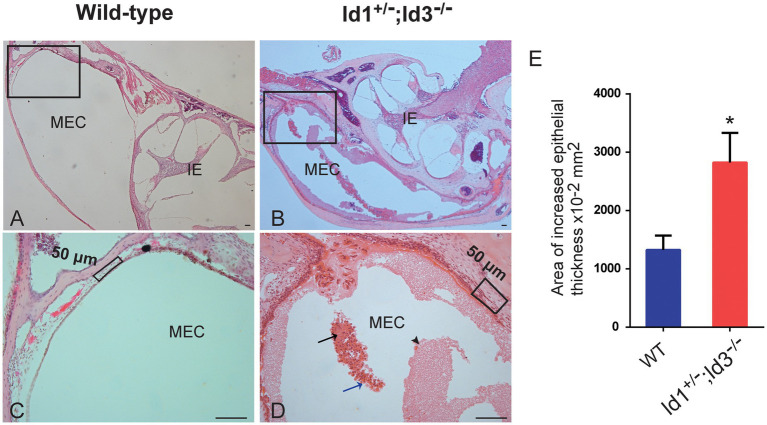
Hematoxylin-Eosin (H&E) staining of histopathology of wild-type and Id1^+/−^; Id3^−/−^ mice. **(A-D)** Representative histology showed obvious inflammatory infiltrations in the MEC of Id1^+/−^; Id3^−/−^ mice **(B,D)** compared to the wild-type mice **(A,C)**. Scale bars: 50 μm. **(A)** The MEC and inner ear (IE) were absent of inflammations. **(C)** The boxed region of **(A)**. **(B)** Panoramic view of the histology showed various inflammations in the MEC. **(D)** At higher magnification, the boxed region in **(B)** revealed detailed view of the cell infiltrations. Exudates of fibrin and blood plasma were evident (black arrowhead). Masses of erythrocytes (black arrow) together with neutrophils (blue arrow) were also detectable in the MEC. **(E)** Comparison of the area of epithelial thickness in MEC between Wild-type mice and Id1^+/−^; Id3^−/−^ mice (*n* = 3 mice in each group). ^*^p < 0.05.

A total of 41 Id1^−/−^; Id3^+/−^ mice, 14 Id1^+/−^; Id3^−/−^ mice and 21 wild-type mice, aged 30 days, were randomly chosen to be screened for otitis media. Otoscopic examination of the Id1^−/−^; Id3^+/−^ mice and Id1^+/−^; Id3^−/−^ mice revealed that 51 and 71% were affected with middle ear fluid, inflammation in the TM and opacification of the TM, respectively. Figure 4 shows representative anatomical images under otoscopy of the ears in Id1^−/−^; Id3^+/−^ and Id1^+/−^; Id3^−/−^ mice. To reveal the middle ear histopathology of Id1^−/−^; Id3^+/−^ and Id1^+/−^; Id3^−/−^ mice, the morphology of the middle ears was analyzed using H&E staining. Histological examination showed obvious inflammatory infiltrates in the tympanic cavity of Id1^−/−^; Id3^+/−^ and Id1^+/−^; Id3^−/−^ mice. Different degrees of effusion appeared in the middle ear cavities of the mutant mice and representative image of Id1^+/−^; Id3^−/−^ mice was shown (Figure 5). A high-magnification view of the tissue showed that inflammatory cells of the effusion content were mainly composed of polymorphonuclear cells. Fibrous proliferation also appeared in the middle ear space in some mice. The middle ear mucosa of Id1^−/−^; Id3^+/−^ and Id1^+/−^; Id3^−/−^ mice generally thickened. The middle ear mucosa of Id1^+/−^; Id3^−/−^ mice was significantly thicker than the wild-type mice (*p* = 0.0101).

## Discussion

In this investigation, we found that middle ear function is affected in Id1 and Id3 mutant mice due to OM, which leads to conductive hearing loss in the affected mice. Because Id1/Id3 double-mutant embryos die at approximately E12 (Lyden et al., 1999), and Id1 and Id3 single-knockout mice have normal hearing (Yan et al., 1997; Lyden et al., 1999; Yokota et al., 1999; data not shown), Id1/Id3 heterozygous (Id1^−/−^; Id3^+/−^ and Id1^+/−^; Id3^−/−^) mice were used to study the combined effect of missing Id1/Id3 alleles.

Auditory brainstem response has been used extensively to assess mouse IE function and also offers a valid, simple physiologic test of mouse middle ear inflammation (MacArthur et al., 2006). Overall, most observed Id1^+/−^; Id3^−/−^ and Id1^−/−^; Id3^+/−^ mice had high ABR thresholds in at least one of the stimulus frequencies (click, 8, 16, and 32 kHz) in at least one ear. At P30, both Id1^+/−^; Id3^−/−^ and Id1^−/−^; Id3^+/−^ mice showed elevated ABR thresholds compared to age-matched littermate controls. However, at P60, ABR thresholds for Id1^−/−^; Id3^+/−^ mice at 16 and 32 kHz no longer showed significant differences compared to the controls. Moreover, at P60, the ABR thresholds of Id1^−/−^; Id3^+/−^ mice were lower than those of the Id1^+/−^; Id3^−/−^ mice for all four frequencies tested. Studies have shown that Id1/Id3 genes have redundant functions, and the loss of one gene function is compensated by another (Lowery et al., 2010). However, based on the ABR data, we can speculate that Id3 might play a slightly greater role in middle ear function. A longitudinal tracking in our study indicated that Id1/Id3 mutant mice from P30 to P120 showed no progression of hearing loss; one explanation is that 4-month-old mice are still relatively young, and the deterioration of hearing loss in mutant mice might show up if we test the mutant mice for extended period, such as until 12 months.

Distortion product of otoacoustic emission is commonly used to evaluate outer hair cell integrity in humans and research animals. Several groups have established this method as another way to evaluate the middle ear conductance and as an indirect measure of conductive hearing loss caused by middle ear dysfunction. This is primarily due to the presence of middle ear effusion, which prevents sound transduction to the inner ear for further processing. Besides, due to lesions in the middle ear, the DPOAE energy cannot be released through the middle ear and detected in the external ear canal. We showed that Id1^+/−^; Id3^−/−^ and Id1^−/−^; Id3^+/−^ mice have much lower DP values compared to littermate controls. If this is not due to outer hair cell dysfunction, then middle ear conductance is very likely to be affected in the mutant mice.

To identify the histopathology of the Id mutant mice that leads to conductive hearing loss, we first examined the gross morphology of the TM. Transparent and balanced TM is observed in the control mice. However, in the mutant mice, bubbles are the most common observation through the TM, which is an indication of middle ear effusion. TM retraction, another common observation, is caused by negative pressure in the middle ear cavity (MEC). In some cases, we observed white patches on the TM, which is usually due to soft tissue calcification, which eventually lead to increased stiffness of the TM and decreased membrane conductance. No TM perforation or missing ossicle bone, particularly the malleus, was observed in the mutant mice. These discoveries exclude the possibility of conductive hearing loss originating from abnormal outer ear canals and TMs.

Next, HE stained inner ear and middle ear cross sections were prepared for more detailed evaluation of the middle ear and inner ear morphology. The mutant mice’s inner ears did not show any abnormalities. For example, there was no hair cell, spiral ganglion neuron loss or dislocation of Reissner’s membrane and no stria vascularis defect. However, different degrees of effusion were present in the middle ear cavities of the mutant mice, which is in consistent with the observation through the TM. Inflammatory cells were also present in the MEC for an extended period, primarily polymorphonuclear cells. This is a major feature of chronic middle ear inflammation. Middle ear epithelia generally thickened in most of the mutant mice, another typical feature of middle ear inflammation. Combining ABR threshold data and histological examination, we found that OM and pathology correlated well with ABR threshold data from the Id1^+/−^; Id3^−/−^ and Id1^−/−^; /Id3^+/−^ and wild-type mice. These data together suggest that Id1/Id3 mutations cause middle ear inflammation, which leads to excess effusion, mucosal thickening and the presence of inflammatory cells in the MEC and the inflammation in middle ear causes conductive hearing loss in Id1/Id3 mutant mice.

Id1 and Id3 are two members of a family of four Id proteins (designated Id1 through Id4) that act as dominant negative inhibitors of bHLH transcription factors (Benezra et al., 1990; Christy et al., 1991; Sun et al., 1991; Riechmann et al., 1994). Id1 and Id3 gene in humans are located on chromosomes 20qll (Idl), lp36.1 (Id3), and they share a very similar genomic organization of exon-intron boundaries within their coding regions (Norton et al., 1998). They are co-expressed temporally and spatially during murine neurogenesis and angiogenesis (Lyden et al., 1999). Id genes are expressed in the otic vesicle’s prosensory domains and are involved in hair cell development through an unknown mechanism (Jones et al., 2006; Kamaid et al., 2010). In the larger picture, Id proteins are crucial for the proper development and function of the immune system (Pan et al., 1999; Miyazaki et al., 2014).

The function of Id proteins associated with the immune system has been well documented in several studies (Miyazaki et al., 2014; Zook and Li, 2018; Han et al., 2019). Lack of Id3 leads to impaired B-cell proliferation and immune responses (Pan et al., 1999). A recent study showed that Id1 favors the differentiation of myeloid-derived suppressor cells (MDSCs), but not dendritic cells (Papaspyridonos et al., 2015). MDSCs are a heterogeneous group of immune cells from the myeloid lineage that suppress immunity against infectious agents. These studies point the possible etiology of OM in Id1/Id3 mutant mice to impaired immune responses. It has been well recognized that a compromised immune system is one of the major contributing factors to OM. We speculate that Id1/Id3 mutations lead to an impaired immune system, which compromises the ability of the mutant mice to fight against middle ear inflammation, leading to continuous presence of effusion, epithelia hyperplasia and inflammatory cells.

Vascular endothelial growth factor (VEGF) is one of the most potent angiogenic factors under inflammatory conditions in the middle ear (Juhn et al., 2008). A previous study used clinical specimens of cholesteatoma in the middle ear to identify a transcription factor that regulate the growth of cholesteatoma. They found Id1 is an essential regulator of VEGF in the cholesteatomal matrix and perimatrix (Fukudome et al., 2013). Some studies have demonstrated that TGF-β, VEGF, or hypoxia-inducible factor (HIF) signaling plays a critical role in the pathogenesis of chronic otitis media in animal models (Tateossian et al., 2009, 2013; Cheeseman et al., 2011; Husseman et al., 2012). As the important differentiation regulators, Id proteins regulate different kinds of cellular processes. More studies need to be done to clarify the underlying mechanism of otitis media in Id1/Id3 mutant mice.

In conclusion, we have shown that the Id1^+/−^; Id3^−/−^ and Id1^−/−^; /Id3^+/−^ mice provide excellent models for studying OM. This model’s susceptibility to OM may be related to the mice’s weakened immune response toward infectious agents. Moreover, the individual variability observed in the Id1^+/−^; Id3^−/−^ and Id1^−/−^; /Id3^+/−^ mouse population may provide a valuable control in future explorations of this model. As such, with this mouse model, we can further elucidate causal relationships between the multiple features of OM and provide an optimal approach to minimizing hearing loss in affected individuals.

## Data Availability Statement

The datasets generated for this study are available on request to the corresponding author.

## Ethics Statement

The experimental protocols were approved by the Case Western Reserve University Animal Care and Use Committee and were in agreement with the National Institutes of Health Guide for the Care and Use of Laboratory Animals.

## Author Contributions

YZ and QZ contributed to the study concept and design. AZ and BL organized the database. TZ performed the statistical analysis and wrote the first draft of the manuscript. BY participated in manuscript revision. All authors contribed to the article and approved the submitted version.

## Conflict of Interest

The authors declare that the research was conducted in the absence of any commercial or financial relationships that could be construed as a potential conflict of interest.

## Publisher’s Note

All claims expressed in this article are solely those of the authors and do not necessarily represent those of their affiliated organizations, or those of the publisher, the editors and the reviewers. Any product that may be evaluated in this article, or claim that may be made by its manufacturer, is not guaranteed or endorsed by the publisher.

## Supplementary Material

Supplementary Figure 1 Sample flow chart of the experiments. A total of 101, Id1^−/−^; Id3^+/−^ mice, 28 Id1^+/−^; Id3^−/−^ mice and 30 wild-type mice were used in this study. ABR test was performed at four time points: P30, P60, P90, and P120. After ABR test, DPOAE measurement was performed at two time points: P30 and P60. After ABR and DPOAE test at P30, otoscopic examination was carried out and then some of the mice were euthanized to conduct H&E staining. *, six mice was from P60.
