# Supplementary figures and images for: Hearing Loss in Id1−/−; Id3+/− and Id1+/−; Id3−/− Mice Is Associated With a High Incidence of Middle Ear Infection (Otitis Media)
Source: Front Genet. 2021 Aug 9;12:508750. doi: 10.3389/fgene.2021.508750 (PMC8381378; doi:10.3389/fgene.2021.508750)

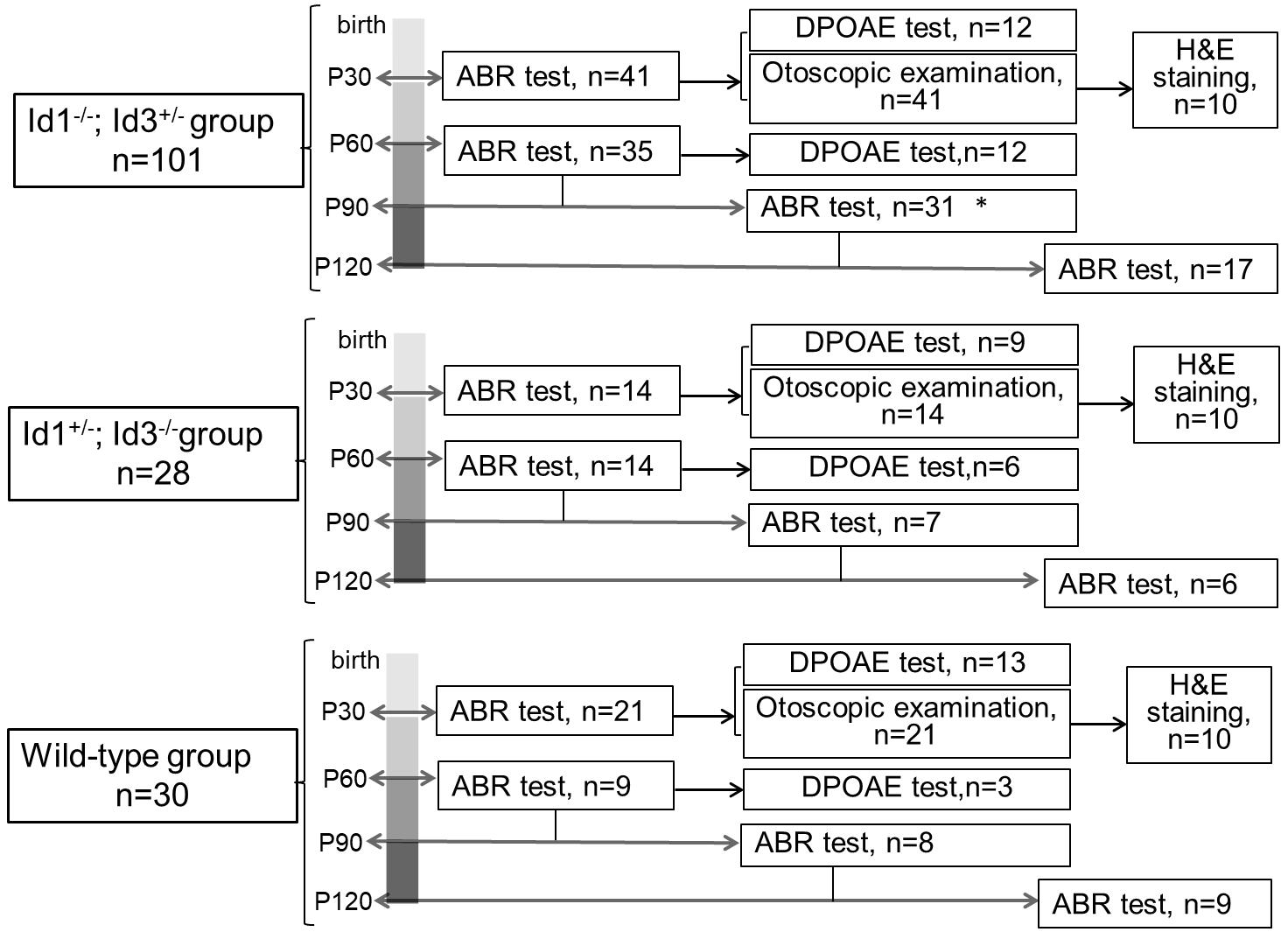

Supplement: Supplementary Figure 1 — Sample flow chart of the experiments. A total of 101, Id1−/−; Id3+/− mice, 28 Id1+/−; Id3−/− mice and 30 wild-type mice were used in this study. ABR test was performed at four time points: P30, P60, P90, and P120. After ABR test, DPOAE measurement was performed at two time points: P30 and P60. After ABR and DPOAE test at P30, otoscopic examination was carried out and then some of the mice were euthanized to conduct H&E staining. *, six mice was from P60. [file Image_1.TIF]
